# Supplementary material for: Sex differences in the association of vitamin D and metabolic risk factors with carotid intima-media thickness in obese adolescents
Source: PLoS One. 2021 Oct 15;16(10):e0258617. doi: 10.1371/journal.pone.0258617 (PMC8519449; doi:10.1371/journal.pone.0258617)
Supplement: S1 Appendix — (DOCX) [file pone.0258617.s001.docx]

**Appendix. Characteristics of obese adolescents who had and had not CIMT measured**

|  | All obese adolescents, n=229 | | |
| --- | --- | --- | --- |
|  | CIMT performed n=156 | Not performed  n=73 | p |
| Mean (SD) age, years | 16.4 (0.7) | 16.2 (0.9) | 0.10 |
| Mean (SD) weight, kg | 87.3 (14.2) | 88.8 (13.2) | 0.43 |
| Mean (SD) height, cm | 162.7 (9.2) | 163.8 (8.9) | 0.41 |
| Mean (SD) BMI, kg/m^2^ | 32.8 (3.5) | 33.0 (3.5) | 0.68 |
| Mean (SD) BMI z-scores | 2.6 (0.4) | 2.7 (0.4) | 0.52 |
| Mean (SD) waist circumference, cm | 93.3 (10.4) | 95.1 (9.9) | 0.21 |
| Mean (SD) waist-to-Height Ratio | 0.57 (0.05) | 0.58 (0.05) | 0.27 |
| Median (Q1;Q3) Vitamin D, ng/mL | 34.1 (17.7;50.3) | 29.1 (16.5;47.3) | 0.23* |
| Median (Q1;Q3) Insulin, μIU/mL | 31.0 (21.4-49.2) | 32.3 (22.6-47.7) | 0.65* |
| Mean (SD) Fasting Plasma Glucose, mg/dL | 86.6 (11.3) | 86 (11.4) | 0.70 |
| Median (Q1;Q3) HOMA-IR | 6.3 (4.1;9.5) | 5.9 (4.2;8.9) | 0.76* |
| Mean (SD) HbA1C, % | 5.2 (0.6) | 5.2 (0.3) | 0.91 |
| Mean (SD) Cholesterol, mg/dL | 175.9 (31.6) | 184.0 (34.3) | 0.10 |
| Mean (SD) LDL-C, mg/dL | 118.9 (29.0) | 124.1 (32.2) | 0.24 |
| Mean (SD) HDL-C, mg/dL | 45.0 (8.9) | 45.7 (9.4) | 0.55 |
| Median (Q1;Q3) Triglyceride, mg/dL | 108.5 (82.3;52.0) | 124 (97.5;182.5) | 0.03* |

*Mann-Whitney-U tests, otherwise independent t tests

LDL-C=low-density lipoprotein cholesterol, HDL-C=high-density lipoprotein cholesterol
