# Supplementary figures and images for: Sex differences in the association of vitamin D and metabolic risk factors with carotid intima-media thickness in obese adolescents
Source: PLoS One. 2021 Oct 15;16(10):e0258617. doi: 10.1371/journal.pone.0258617 (PMC8519449; doi:10.1371/journal.pone.0258617)

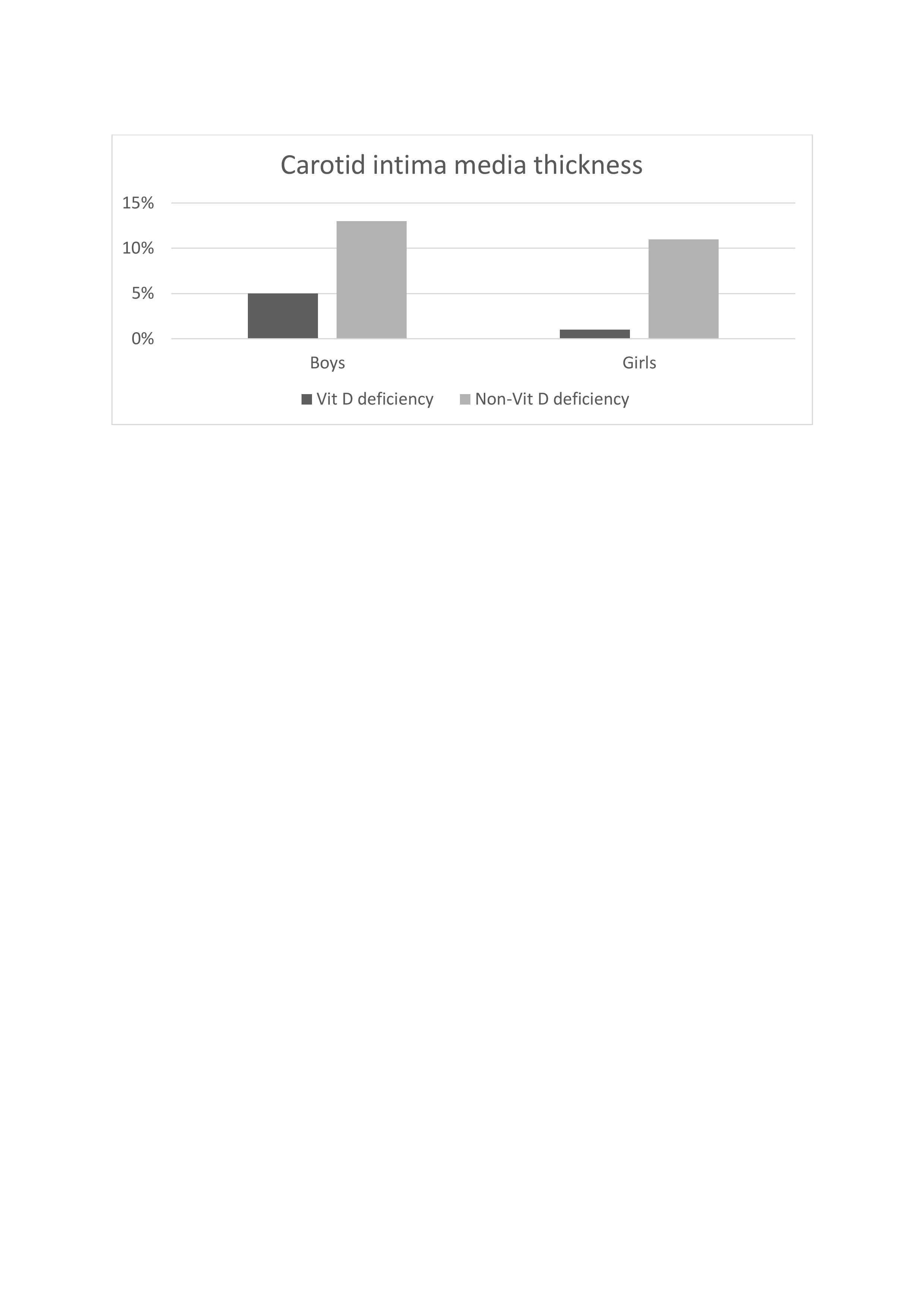

Supplement: S1 Fig — (TIFF) [file pone.0258617.s002.tiff]
