# Supplementary material for: Sex differences in the association of vitamin D and metabolic risk factors with carotid intima-media thickness in obese adolescents
Source: PLoS One. 2021 Oct 15;16(10):e0258617. doi: 10.1371/journal.pone.0258617 (PMC8519449; doi:10.1371/journal.pone.0258617)
Supplement: Attachment — Submitted filename: Response to Reviewers.docx [file pone.0258617.s004.docx]

RESPONSE TO REVIEWERS

Thank you for the constructive questions and feedbacks. The responses are written in blue.

Thank you for submitting your manuscript to PLOS ONE. After careful consideration, we feel that it has merit but does not fully meet PLOS ONE’s publication criteria as it currently stands. Therefore, we invite you to submit a revised version of the manuscript that addresses the points raised during the review process.

While the study had a lot of merit, there were a number of issues that need to be addressed.  First their were significant grammatically errors throughout the text. Second, the data presented in the tables is unclear (what is being presented, skewed data is not indicated, and what was being compared). Third, why was Vitamin D not examined as a continuous variable? Also, from the raw data it appears that blood pressure was measured, why was this not included in the analysis? Lastly, as you did not find a association between vitD and CIMT, why was the analysis not extended to look at vitamin D to other metabolic risk factors measured.

Please submit your revised manuscript by Sep 13 2020 11:59PM. If you will need more time than this to complete your revisions, please reply to this message or contact the journal office at plosone@plos.org. When you're ready to submit your revision, log on to https://www.editorialmanager.com/pone/ and select the 'Submissions Needing Revision' folder to locate your manuscript file.

Please include the following items when submitting your revised manuscript:

- A rebuttal letter that responds to each point raised by the academic editor and reviewer(s). You should upload this letter as a separate file labeled 'Response to Reviewers'.
- A marked-up copy of your manuscript that highlights changes made to the original version. You should upload this as a separate file labeled 'Revised Manuscript with Track Changes'.
- An unmarked version of your revised paper without tracked changes. You should upload this as a separate file labeled 'Manuscript'.

If you would like to make changes to your financial disclosure, please include your updated statement in your cover letter. Guidelines for resubmitting your figure files are available below the reviewer comments at the end of this letter.

If applicable, we recommend that you deposit your laboratory protocols in protocols.io to enhance the reproducibility of your results. Protocols.io assigns your protocol its own identifier (DOI) so that it can be cited independently in the future. For instructions see: <http://journals.plos.org/plosone/s/submission-guidelines#loc-laboratory-protocols>

We look forward to receiving your revised manuscript.

Kind regards,

Jonathan M Peterson, Ph.D.
Academic Editor
PLOS ONE

Journal Requirements:

When submitting your revision, we need you to address these additional requirements.

1. Please ensure that your manuscript meets PLOS ONE's style requirements, including those for file naming. The PLOS ONE style templates can be found at

https://journals.plos.org/plosone/s/file?id=wjVg/PLOSOne_formatting_sample_main_body.pdf and

https://journals.plos.org/plosone/s/file?id=ba62/PLOSOne_formatting_sample_title_authors_affiliations.pdf

Response to the Reviewer:

Thank you, noted.

2. We suggest you thoroughly copyedit your manuscript for language usage, spelling, and grammar. If you do not know anyone who can help you do this, you may wish to consider employing a professional scientific editing service.  

Whilst you may use any professional scientific editing service of your choice, PLOS has partnered with both American Journal Experts (AJE) and Editage to provide discounted services to PLOS authors. Both organizations have experience helping authors meet PLOS guidelines and can provide language editing, translation, manuscript formatting, and figure formatting to ensure your manuscript meets our submission guidelines. To take advantage of our partnership with AJE, visit the AJE website (http://learn.aje.com/plos/) for a 15% discount off AJE services. To take advantage of our partnership with Editage, visit the Editage website (www.editage.com) and enter referral code PLOSEDIT for a 15% discount off Editage services.  If the PLOS editorial team finds any language issues in text that either AJE or Editage has edited, the service provider will re-edit the text for free.

Upon resubmission, please provide the following:

●      The name of the colleague or the details of the professional service that edited your manuscript

●      A copy of your manuscript showing your changes by either highlighting them or using track changes (uploaded as a *supporting information* file)

●      A clean copy of the edited manuscript (uploaded as the new *manuscript* file)

Response to the Reviewer:

The manuscript had been checked for the grammars and spellings by:

Erik Christopher Hookom, BA, Med, TEFL

Office of Research and Publication (ORP)

Faculty of Medicine, Public Health, and Nursing Universitas Gadjah Mada

Administration Building 2^nd^ Floor

Phone: 0274 560300 ext 205

Email: [orp.fm@ugm.ac.id](mailto:orp.fm@ugm.ac.id); echookom@gmail.com

A copy of manuscript showing track changes and a clean copy of the edited manuscript have been uploaded as a supporting information file and the new manuscript file, respectively.

3. In your Methods section, please provide additional information about the participant recruitment method and the demographic details of your participants. Please ensure you have provided sufficient details to replicate the analyses such as: a) the recruitment date range (month and year), b) a description of any inclusion/exclusion criteria that were applied to participant recruitment, c) a table of relevant demographic details, d) a statement as to whether your sample can be considered representative of a larger population, e) a description of how participants were recruited, and f) descriptions of where participants were recruited and where the research took place.

Response to the Reviewer:

Thank you for the feedbacks. The recruitment process was done by screening for adolescents with obesity in seven public and three private high schools in the city of Yogyakarta, a city in the Southern part of Java, Indonesia. The participants were recruited at their schools and we screened 4268 students from 10 schools in Yogyakarta using three body mass index reference cut-off points: World Health Organization (WHO), Centre for Disease Control and Prevention (CDC), and International Obesity Task Force (IOTF). The screening was done from January to February 2016. We identified 298 (7%) adolescents classified as obese based on all those three obesity criteria. Of those, 229 (76.8%) obese adolescents agreed to participate in the next step of the study, i.e. assessment of their metabolic risk. The blood collection was taken at their schools, while the assessment of carotid intima media thickness was performed at the Dr Sardjito Hospital, Yogyakarta. These data collection was done from March to October 2016.

Inclusion criteria were obese children aged 15 to less than 18 years. We excluded children with diabetes mellitus, renal disease, cardiovascular disease, any history of any systemic disease or history of current steroid use. This study included 156 (out of 229) obese adolescents who had data on CIMT. We considered that the sample of this study could be considered representative of a larger population.

There were no differences in metabolic risk parameters between adolescents who had their CIMT measured versus those who had not (Appendix).

4. Thank you for stating in your Funding Statement:

 "The study was partly funded by the Indonesian Pediatric Society and Frisian Flag Indonesia (355/Legal/FFI/XII/2014)".

i) Please provide an amended statement that declares *all* the funding or sources of support (whether external or internal to your organization) received during this study, as detailed online in our guide for authors at http://journals.plos.org/plosone/s/submit-now.  Please also include the statement “There was no additional external funding received for this study.” in your updated Funding Statement.

Response to the Reviewer:

Thank you for the feedback. Regarding the funding of this study, we declare that the study was funded by the Indonesian Pediatric Society and Frisian Flag Indonesia (355/Legal/FFI/XII/2014). There was no additional external funding received for this study.

ii) Please include your amended Funding Statement within your cover letter. We will change the online submission form on your behalf.

Response to the Reviewer:

Thank you, the amended Funding Statement has been included in the cover letter.

5. Please include captions for your Supporting Information files at the end of your manuscript, and update any in-text citations to match accordingly. Please see our Supporting Information guidelines for more information: http://journals.plos.org/plosone/s/supporting-information.

Response to the Reviewer:

Done

Additional Editor Comments (if provided):

While the study had a lot of merit, there were a number of issues that need to be addressed. First their were significant grammatically errors throughout the text. Second, the data presented in the tables is unclear (what is being presented, skewed data is not indicated, and what was being compared). Third, why was Vitamin D not examined as a continuous variable? Also, from the raw data it appears that blood pressure was measured, why was this not included in the analysis? Lastly, as you did not find a association between vitD and CIMT, why was the analysis not extended to look at vitamin D to other metabolic risk factors measured.

While the study had a lot of merit, there were a number of issues that need to be addressed.  First their were significant grammatically errors throughout the text.

Response to the Editor:

The manuscript had been checked grammatically by a professional scientific editing service and this has been stated in the cover letter.

Second, the data presented in the tables is unclear (what is being presented, skewed data is not indicated, and what was being compared).

Response to the Reviewer:

Thank you for the feedback. We have added explanation on the dependent and independent variables on the Tables and the skewed data have been clearly indicated.

Table 1 describes the characteristics of obese adolescents from Yogyakarta stratified by sex. Normally distributed and skewed data are presented as mean (SD) and median (Q1;Q3), respectively. Normally distributed data are compared using independent sample t-test, while skewed data are compared using independent-samples Mann-Whitney U test. Categorical data are presented as n (%) and compared using chi-square tests unless otherwise indicated.

Table 2 describes sex differences in the association of vitamin D and metabolic risk factors with carotid intima-media thickness in obese adolescents from Yogyakarta, Indonesia.

The dependent variable was CIMT as a marker of early vascular impairment. The independent variables were vitamin D and other metabolic risk factors including waist-to-hip ratio (marker of abdominal obesity), blood glucose, HbA1c (impaired glucose metabolism), fasting insulin, HOMA-IR (insulin resistance), LDL and HDL cholesterol, triglyceride (impaired lipid metabolism), blood pressure, smoking and family history of hypertension.

Table 2 shows that overall, elevated HOMA-IR, total cholesterol, LDL-cholesterol and triglyceride levels were associated with greater odds of elevated CIMT. The associations persisted after adjustment for age, sex and second-hand smoke exposure. However, after the data were analysed within each sex, similar results were only observed in boys. None of the risk factors were associated with CIMT in girls.

Third, why was Vitamin D not examined as a continuous variable?

Response to the Reviewer:

Thank you for the feedback. We actually are interested in looking at the clinical consequences of deficiency or insufficiency cut-offs of vitamin D status with the carotid intima media thickness as a measure of vascular thickness (a subclinical atherosclerosis in children). We used different cut off: Vitamin D level ≤30 ng/mL or ≤20 ng/mL. A categorisation of continuous data of vitamin D level was used in this study because it is easier to interpret and more feasible to present in clinical practice. It is helpful to label children and adolescents as having deficient, insufficient or normal vitamin D level. We hope that this paper is easier for clinicians to read and understand in order to make clinical decision making where decisions typical are categorical in nature.

Also, from the raw data it appears that blood pressure was measured, why was this not included in the analysis?

Response to the Editor:

We have included the analysis of blood pressure (as one of independent variables) and CIMT (as a dependent variable or outcome) at Table 2.

Lastly, as you did not find a association between vitD and CIMT, why was the analysis not extended to look at vitamin D to other metabolic risk factors measured.

Response to the Editor:

Thank you for the feedback. It seems like the introduction section of the paper was not clear enough to highlight why we are interested in looking at CIMT as predicted by vitamin D status independent of other predictors of CIMT.

A paragraph has been added in the Introduction to further clarify the aim of the study.

Carotid intima-media thickness (CIMT) is regarded as a reliable marker of subclinical atherosclerosis since increased CIMT can reflect an increase in arterial wall thickness including in children and adolescent (Atabek et al, 2014). Increased CIMT has been predicted in children with obesity, familial hypercholesterolemia, type 1 diabetes, hypertension (Giladini et al, 2011) and vitamin D deficiency (Atabek et al, 2014). In this study, we are particularly interested in looking at CIMT as predicted by vitamin D status independent of other predictors of CIMT.

The dependent variable was CIMT as a marker of early vascular impairment. The independent variables were vitamin D and other metabolic risk factors including waist-to-hip ratio (marker of abdominal obesity), blood glucose, HbA1c (impaired glucose metabolism), fasting insulin, HOMA-IR (insulin resistance), LDL and HDL cholesterol, triglyceride (impaired lipid metabolism), blood pressure, smoking and family history of hypertension.

The association of vitamin D and other metabolic risk factors with CIMT was explored using logistic regression and presented as odds ratio (95% confidence interval). Each factor was tested in a separate regression model. Models that were significantly associated with elevated CIMT, were re-tested with adjustment for age, sex and secondary smoke exposures. All analyses were performed on a sample as a whole and also stratified by sex based on biological differences in body fat accumulation and potentially vitamin D levels between boys and girls.

[Note: HTML markup is below. Please do not edit.]

Reviewers' comments:

Reviewer's Responses to Questions

**Comments to the Author**

1. Is the manuscript technically sound, and do the data support the conclusions?

The manuscript must describe a technically sound piece of scientific research with data that supports the conclusions. Experiments must have been conducted rigorously, with appropriate controls, replication, and sample sizes. The conclusions must be drawn appropriately based on the data presented.

Reviewer #1: Yes

2. Has the statistical analysis been performed appropriately and rigorously?

Reviewer #1: No

3. Have the authors made all data underlying the findings in their manuscript fully available?

The [PLOS Data policy](http://www.plosone.org/static/policies.action#sharing) requires authors to make all data underlying the findings described in their manuscript fully available without restriction, with rare exception (please refer to the Data Availability Statement in the manuscript PDF file). The data should be provided as part of the manuscript or its supporting information, or deposited to a public repository. For example, in addition to summary statistics, the data points behind means, medians and variance measures should be available. If there are restrictions on publicly sharing data—e.g. participant privacy or use of data from a third party—those must be specified.

Reviewer #1: Yes

4. Is the manuscript presented in an intelligible fashion and written in standard English?

PLOS ONE does not copyedit accepted manuscripts, so the language in submitted articles must be clear, correct, and unambiguous. Any typographical or grammatical errors should be corrected at revision, so please note any specific errors here.

Reviewer #1: Yes

5. Review Comments to the Author

Please use the space provided to explain your answers to the questions above. You may also include additional comments for the author, including concerns about dual publication, research ethics, or publication ethics. (Please upload your review as an attachment if it exceeds 20,000 characters)

Reviewer #1: Paper has lot of grammatical errors and flow is very limited
Need to assess high sensitivity CRP/?cardiac risk/LVMI and 24 hr ABPM or BP and would have correlated these parameters with those variables
Vitamin d level should be correlated with these parameters as we do not know what level is optimal one
urine albumin to creatinine ratio should reassesses
I will assess height and growth parameters 'also
need to develop or assess cardiovascular risk score like in adult to assess risk prediction
we should look into family history
It is association not causation

Need to assess high sensitivity CRP/?cardiac risk/LVMI and 24 hr ABPM or BP and would have correlated these parameters with those variables. Vitamin d level should be correlated with these parameters as we do not know what level is optimal one

Response to the Reviewer:

Thank you for the feedback. In this study, we particularly interested in looking at CIMT as predicted by vitamin D status independent of other predictors of CIMT. We are interested in looking at the clinical consequences of deficiency or insufficiency cut-offs of vitamin D status with the carotid intima media thickness as a measure of vascular thickness (a subclinical atherosclerosis in children).

We did not have data on hsCRP. But we did analyses on the association between CIMT (as an outcome) and vitamin D and other cardiovascular and metabolic risk factors including abdominal obesity (waist to hip ratio), blood glucose, HbA1c (glucose metabolism impairment), fasting insulin, HOMA-IR (insulin resistance), LDL and HDL cholesterol, triglyceride (dyslipidaemia), blood pressure, smoking, and family history of hypertension. We have added a paragraph in the Method section to clarify this.

We have added analysis on blood pressure and CIMT in the Table 2. But we did not include the analysis of LVMI in this study because we will do in another paper.

urine albumin to creatinine ratio should reassesses

Response to the Reviewer:

Thank you for the feedback. We did not have data on albumin and creatinine ratio.

I will assess height and growth parameters 'also

Response to the Reviewer:

Thank you for the feedback. We have analysis the height in term of waist-to-hip ratio between boys and girls in the Table 1 and the association between high waist-to-hip ratio with CIMT in the Table 2. But we did not analyse the association between growth with CIMT. We did not have the data of growth because of the nature of cross sectional study design.

need to develop or assess cardiovascular risk score like in adult to assess risk prediction
we should look into family history

Response to the Reviewer:

Thank you for the feedback. In this study we did not perform a cardiovascular risk score, we only assessed whether vitamin D and other metabolic risk factors predicted the increased CIMT without creating a score. But we have included a family history of hypertension as one of predictors of increased CIMT in this study.

It is association not causation

Response to the Reviewer:

Thank you, noted.

6. PLOS authors have the option to publish the peer review history of their article ([what does this mean?](https://journals.plos.org/plosone/s/editorial-and-peer-review-process#loc-peer-review-history)). If published, this will include your full peer review and any attached files.


If you choose “no”, your identity will remain anonymous but your review may still be made public.


**Do you want your identity to be public for this peer review?** For information about this choice, including consent withdrawal, please see our [Privacy Policy](https://www.plos.org/privacy-policy).

Reviewer #1: No

[NOTE: If reviewer comments were submitted as an attachment file, they will be attached to this email and accessible via the submission site. Please log into your account, locate the manuscript record, and check for the action link "View Attachments". If this link does not appear, there are no attachment files.]

While revising your submission, please upload your figure files to the Preflight Analysis and Conversion Engine (PACE) digital diagnostic tool, <https://pacev2.apexcovantage.com/>. PACE helps ensure that figures meet PLOS requirements. To use PACE, you must first register as a user. Registration is free. Then, login and navigate to the UPLOAD tab, where you will find detailed instructions on how to use the tool. If you encounter any issues or have any questions when using PACE, please email PLOS at [figures@plos.org](mailto:figures@plos.org). Please note that Supporting Information files do not need this step.
