# Supplementary material for: Sex differences in the association of vitamin D and metabolic risk factors with carotid intima-media thickness in obese adolescents
Source: PLoS One. 2021 Oct 15;16(10):e0258617. doi: 10.1371/journal.pone.0258617 (PMC8519449; doi:10.1371/journal.pone.0258617)
Supplement: Attachment — Submitted filename: Response to Reviewer.docx [file pone.0258617.s005.docx]

Response to Reviewer

Thank you for the constructive questions and feedbacks. The responses are written in blue.

Additional Editor Comments (if provided):

First I would like to apologize for the long delay, it was unfortunately as we had significant issues securing a reviewer for the manuscript.
I agree with the new reviewers comments that the manuscript is much improved, but analysis of VitD as a continuous variable should also be included. Regarding the table legends, it is still unclear what is being compared. For table 1 the p value appears to be reporting significance comparing boys and girls? However, the p-values reported for table 2 are difficult to discern and are not explained clearly in the legend.

Thank you. The analysis of vitamin D levels as a continuous variable has been provided below:

Table 2. Correlations between of vitamin D levels and other metabolic risk factors to predict carotid intima-media thickness in obese adolescents

|  | **Boys (n=87)** | | **Girls (n=69)** | |
| --- | --- | --- | --- | --- |
|  | ρ | p | ρ | p |
| Age, years | -0.027 | 0.801 | 0.044 | 0.717 |
| Waist-to-hip ratio | 0.119 | 0.273 | -0.144 | 0.237 |
| Systolic blood pressure, mmHg | 0.078 | 0.472 | 0.094 | 0.444 |
| Diastolic blood pressure, mmHg | -0.032 | 0.767 | 0.104 | 0.396 |
| Vitamin D, ng/mL | 0.077 | 0.481 | 0.163 | 0.182 |
| Fasting insulin, mg/dL | 0.104 | 0.339 | -0.127 | 0.303 |
| Fasting blood glucose, mg/dL | -0.139 | 0.198 | 0.082 | 0.501 |
| HOMA-IR | 0.088 | 0.420 | -0.125 | 0.309 |
| HbA1C, g/dL | -0.015 | 0.889 | 0.114 | 0.349 |
| Total cholesterol, mg/dL | 0.316 | 0.003 | -0.029 | 0.814 |
| LDL cholesterol, mg/dL | 0.337 | 0.001 | -0.012 | 0.920 |
| HDL cholesterol, mg/dL | -0.085 | 0.433 | -0.132 | 0.279 |
| Triglyceride, mg/dL | 0.152 | 0.160 | 0.105 | 0.392 |

LDL=low density lipoprotein; HDL=high density lipoprotein; HOMA-IR = homeostatic model assessment of insulin resistance; HbA1C = glycated hemoglobin.

We found no correlation between vitamin D levels and CIMT, but we found a positive correlation between LDL and total cholesterols with CIMT.

We also provide Table 3 showing the association of vitamin D levels and other metabolic risk factors to predict carotid intima-media thickness between boy and girl obese adolescents. Normally distributed continuous data are compared using independent sample t-test, while skewed data are compared using independent-samples Mann-Whitney U test. Categorical data are presented as n (%) and compared using chi-square tests unless otherwise indicated.

p value in the previous table 1 reported the significance comparing boys and girls.

Odds ratio with its 95% CI and p value in the previous table 2 (now as Table 4) reported the significance of association of vitamin D levels and other metabolic risk factors to predict carotid intima-media thickness comparing boys and girls using logistic regression analysis.

[Note: HTML markup is below. Please do not edit.]

Reviewers' comments:

Reviewer's Responses to Questions

**Comments to the Author**

1. If the authors have adequately addressed your comments raised in a previous round of review and you feel that this manuscript is now acceptable for publication, you may indicate that here to bypass the “Comments to the Author” section, enter your conflict of interest statement in the “Confidential to Editor” section, and submit your "Accept" recommendation.

Reviewer #2: (No Response)

2. Is the manuscript technically sound, and do the data support the conclusions?

The manuscript must describe a technically sound piece of scientific research with data that supports the conclusions. Experiments must have been conducted rigorously, with appropriate controls, replication, and sample sizes. The conclusions must be drawn appropriately based on the data presented.

Reviewer #2: Partly

3. Has the statistical analysis been performed appropriately and rigorously?

Reviewer #2: No

4. Have the authors made all data underlying the findings in their manuscript fully available?

The [PLOS Data policy](http://www.plosone.org/static/policies.action#sharing) requires authors to make all data underlying the findings described in their manuscript fully available without restriction, with rare exception (please refer to the Data Availability Statement in the manuscript PDF file). The data should be provided as part of the manuscript or its supporting information, or deposited to a public repository. For example, in addition to summary statistics, the data points behind means, medians and variance measures should be available. If there are restrictions on publicly sharing data—e.g. participant privacy or use of data from a third party—those must be specified.

Reviewer #2: Yes

5. Is the manuscript presented in an intelligible fashion and written in standard English?

PLOS ONE does not copyedit accepted manuscripts, so the language in submitted articles must be clear, correct, and unambiguous. Any typographical or grammatical errors should be corrected at revision, so please note any specific errors here.

Reviewer #2: No

6. Review Comments to the Author

Please use the space provided to explain your answers to the questions above. You may also include additional comments for the author, including concerns about dual publication, research ethics, or publication ethics. (Please upload your review as an attachment if it exceeds 20,000 characters)

Reviewer #2: The carotid intima-media thickness (CIMT) is already well studied in children and adolescents, including assessing the relationship with low levels of vitamin D 25-OH.
The present study provides data from Indonesian adolescents, which is important to verify possible differences in this specific population.
The grammatical revision of the text really improved its quality. However, some aspects remain deficient:
1. Although the analysis of vitamin D as a categorical variable had been justified, it would be important, which is not so difficult, also analyze it as a continuous variable. Even if the result is negative, the analysis will be more complete. After all, the title, and the entire text focus on vitamin D. Otherwise, I suggest expanding the discussion to the other parameters evaluated, drawing attention to the positive findings. (Osika W, Dangardt F, Montgomery SM, Volkmann R, Gan LM, Friberg P. Sexual differences in the intimate peripheral artery, mean and thickness of the intimate media in children and adolescents. Atherosclerosis. 2009 March; 203 (1): 172-7 .)

Response to the Reviewer:

Thank you. The analysis of vitamin D levels as a continuous variable has been provided in the new version of Table 2 below:

Table 2. Correlations between of vitamin D levels and other metabolic risk factors to predict carotid intima-media thickness in obese adolescents

|  | **Boys (n=87)** | | **Girls (n=69)** | |
| --- | --- | --- | --- | --- |
|  | ρ | p | ρ | p |
| Age, years | -0.027 | 0.801 | 0.044 | 0.717 |
| Waist-to-hip ratio | 0.119 | 0.273 | -0.144 | 0.237 |
| Systolic blood pressure, mmHg | 0.078 | 0.472 | 0.094 | 0.444 |
| Diastolic blood pressure, mmHg | -0.032 | 0.767 | 0.104 | 0.396 |
| Vitamin D, ng/mL | 0.077 | 0.481 | 0.163 | 0.182 |
| Fasting insulin, mg/dL | 0.104 | 0.339 | -0.127 | 0.303 |
| Fasting blood glucose, mg/dL | -0.139 | 0.198 | 0.082 | 0.501 |
| HOMA-IR | 0.088 | 0.420 | -0.125 | 0.309 |
| HbA1C, g/dL | -0.015 | 0.889 | 0.114 | 0.349 |
| Total cholesterol, mg/dL | 0.316 | 0.003 | -0.029 | 0.814 |
| LDL cholesterol, mg/dL | 0.337 | 0.001 | -0.012 | 0.920 |
| HDL cholesterol, mg/dL | -0.085 | 0.433 | -0.132 | 0.279 |
| Triglyceride, mg/dL | 0.152 | 0.160 | 0.105 | 0.392 |

LDL=low density lipoprotein; HDL=high density lipoprotein; HOMA-IR = homeostatic model assessment of insulin resistance; HbA1C = glycated hemoglobin.

We found no correlation between vitamin D and CIMT, but we found a positive correlation between LDL and total cholesterols with CIMT.

We also provide Table 3 showing the association of vitamin D levels and other metabolic risk factors to predict carotid intima-media thickness between boy and girl obese adolescents. Normally distributed continuous data are compared using independent sample t-test, while skewed data are compared using independent-samples Mann-Whitney U test. Categorical data are presented as n (%) and compared using chi-square tests unless otherwise indicated.

The new table 4 showed association of vitamin D levels and other metabolic risk factors to predict carotid intima-media thickness comparing obese adolescent boys and girls using logistic regression analysis.

2. Verification of normality uses the Kolmogorov-Smirnov test. In the text, “Smirnov” was replaced by a brand of vodka.

Thank you, we have revised accordingly.

3. It is necessary to exclude the form of data presentation in the first columns, leaving only the name of the variable and the unit. The current format is redundant (the information is already described below the tables) and makes it difficult to understand.

Thank you, we have revised accordingly as suggested.

7. PLOS authors have the option to publish the peer review history of their article ([what does this mean?](https://journals.plos.org/plosone/s/editorial-and-peer-review-process#loc-peer-review-history)). If published, this will include your full peer review and any attached files.


If you choose “no”, your identity will remain anonymous but your review may still be made public.


**Do you want your identity to be public for this peer review?** For information about this choice, including consent withdrawal, please see our [Privacy Policy](https://www.plos.org/privacy-policy).

Reviewer #2: No
